# Evaluation of Surface Roughness Reduction in TPU 95A Samples Using Ferromagnetic Liquid Machining

**DOI:** 10.3390/ma18214939

**Published:** 2025-10-29

**Authors:** Natalia Kowalska, Slawomir Blasiak, Michał Skrzyniarz, Paweł Szczygieł, Wiktor Szot, Mateusz Rudnik

**Affiliations:** Faculty of Mechatronics and Mechanical Engineering, Kielce University of Technology, Tysiąclecia Państwa Polskiego 7 Ave., 25-314 Kielce, Poland; sblasiak@tu.kielce.pl (S.B.); mskrzyniarz@tu.kielce.pl (M.S.); pszczygiel@tu.kielce.pl (P.S.); wszot@tu.kielce.pl (W.S.); mrudnik@tu.kielce.pl (M.R.)

**Keywords:** 3D printing, MEX technology, finishing

## Abstract

Additive manufacturing technologies are characterised by the capability to produce components with complex geometries that are difficult to achieve using conventional methods. Despite the wide range of available materials and additive manufacturing processes, fulfilling design requirements related to surface structure parameters remains a considerable challenge. This paper presents the findings of an investigation into the influence of abrasive treatment of a ferromagnetic fluid on the surface roughness of MEX-printed samples. The samples were fabricated using TPU 95A material. The abrasive medium employed in the study comprised carbonyl iron and silicon carbide. A dedicated tool was designed for the experiments, incorporating neodymium magnets arranged in four asymmetrically distributed slots. The proposed tool represents an unconventional approach in comparison with existing practices. Tests were conducted in three measurement series—B, C, and D—while series A served as the control group. Analysis of the experimental results revealed that, for the parameters Sp (height of the highest apex) and Sz (maximum height, defined as the sum of Sp and Sv, representing the height of the highest apex and the maximum pit depth, respectively), the most significant reduction in parameter values was observed for series D.

## 1. Introduction

Additive technologies are a group of manufacturing processes considered to be one of the achievements of the Industrial Revolution of the 20th century. The term refers to technologies that build a model in a layer-by-layer fashion [1]. Originally, additive manufactured models served as prototypes, but with the development of materials and improvements in manufacturing processes, additive technologies have been introduced into a variety of industries such as medicine, engineering, and art [2].

The additive manufacturing process can be divided into the following scheme: design and modelling, convert to STL, slicing, 3D printing and post-processing [3].

Fused deposition modelling (FDM) technology belongs to the MEX (material extrusion) category described in ISO/ASTM DIS 52900 [4]. The MEX process involves extrusion of a thermoplastic material through a printer tool (hotend/nozzle/extruder) in the form of a single filament. The material is applied according to a numerically set path. Thermoplastic materials used in the manufacturing process include, acrylonitrile butadiene styrene (ABS), polylactic acid (PLA), polycarbonate (PC), as well as composite materials [5,6], and in specialised printer models, it is possible to print from materials such as concrete or metal [7].

Due to the nature of the manufacturing process as well as the limitations of the apparatus used (among other things, the diameter of the nozzle through which the material is extruded), components manufactured using MEX technology are characterised by high roughness and low accuracy of the final models [8]. The following parameters have a large impact on the dimensional accuracy of the shape: layer height, printing temperature, and printing speed. The problem of optimising model manufacturing is described in the article [9], which uses the grey-wolf optimisation algorithm to select the parameters layer height and printing temperature with the lowest roughness expressed by the Ra parameter. However, the technological parameters also affect the fabrication time; choosing parameters (low layer height, etc.) significantly increases the printing time [10]. In order to improve the quality of the surface layer, it is necessary to perform finishing operations involving chemical, thermal, and machining treatments. Chemical treatment is carried out using chemical reagents that are solvents for the material in question. An example of chemical treatment was realised in the article in [11] using acetone. The samples were made of ABS material and then placed in a chamber partially filled with a pure acetone solution; the vapourisation process of the sample lasted from 15 to 135 min. The described test showed a decrease in the Ra parameter value of up to 98%. However, prolonged exposure of the model to the chemical solution can lead to surface deformation [12]. Alternatively, incrementally produced models are machined to reduce surface roughness parameters. An example of such a process is presented in the article in [13], in which two models of specimens printed at an angle of 0 to 90° relative to the working platform were prepared from PLA material using FDM technology. The surface roughness of the specimens was measured at selected points on the surface of the specimens, followed by machining and repeating the measurements at predetermined points. The authors concluded by recognising the potential of combining additive manufacturing processes with machining, acting as a finishing treatment, due to the possibility of obtaining better surface quality and lower tolerances.

The finishing of additively manufactured models is mainly carried out by machining and chemical processing. Specialised processes are also used, such as abrasive processing with glass beads or abrasive particles combined with a magnetic fluid. This process involves the use of a magnetological fluid, based on a suspension of ferromagnetic particles in a carrier fluid [14]. This fluid changes its stiffness as the magnetic field strength around the tool changes. There are two types of fluids that react to changes in magnetic fields: ferromagnetic and magnetorheological. Ferromagnetic fluids (abbreviated FF) are made from particles with a diameter of several nanometres. Magnetorheological fluids (abbreviated as MRF), on the other hand, contain particles measuring several micrometres. The interaction of the magnetic field affects their rheological properties (dynamic viscosity, yield point). The range of changes in the properties of the fluid is a function of many parameters; in particular, the volume fraction of magnetic particles in the fluid, the magnetic properties of the particle material, the size and shape of the particles, and the parameters of the magnetic field. The range of variability in properties is much greater in MRF fluids than in FF fluids. The MR (magnetorheological fluid) process is used for precision finishing of surfaces, particularly curved surfaces [15]. One variant of magnetorheological finishing is the BEMRF (magnetorheological ball end finishing) process [16]. In this process, the most important component is a vertical spindle with an electromagnet mounted; inside the spindle is a tube through which fluid is fed. As a result of the paramagnetic effect and the rotation performed by the spindle, a ball of MR fluid and abrasive particles is formed at the end of the tool nozzle. As a result of the movement of the fluid, the abrasive particles abrade the surfaces of the model. Machining with the BEMRF can be used to machine additive manufactured parts, as shown in [17], achieving a reduction in surface roughness from Ra = 20 μm to Ra = 81 nm.

The purpose of this article is to investigate the processing of additively manufactured samples made from TPU 95A HF material using a ferromagnetic fluid. The study introduces a novel tool that has not been employed in the conventional BERMF process. The results presented herein constitute the first stage in the development of a fully functional tool with optimised processing parameters.

## 2. Materials and Methods

The finishing process using ferromagnetic fluid consisted of the following steps: designing the sample in CAD 2023 software (SOLIDWORKS, Dassault Systèmes SolidWorks Corporation, Waltham, MA, USA) and saving the STL file, printing out the models, which formed three measurement series, carrying out surface roughness measurements before machining, carrying out finishing, and repeating the measurements after machining.

**Figure 1:**
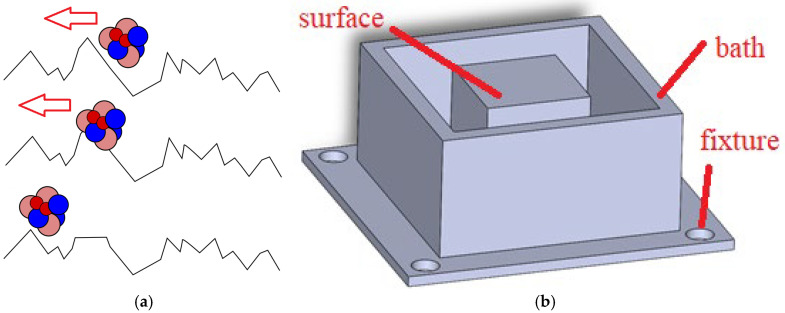
(**a**) Scheme of operation, (**b**) sample design.

Figure 1a shows a simplified schematic of machining with ferromagnetic fluid; the mixed abrasive and fluid particles abrade primarily the tops of the sample profile elevations.

The prepared samples (Figure 1b) consisted of three elements: a bath, a fixture, and the actual machined surface, which is a 30 mm × 30 mm × 30 mm cube. Due to the planned different cases, the samples were printed with the tub representing the object, allowing it to be filled with fluid.

**Figure 2:**
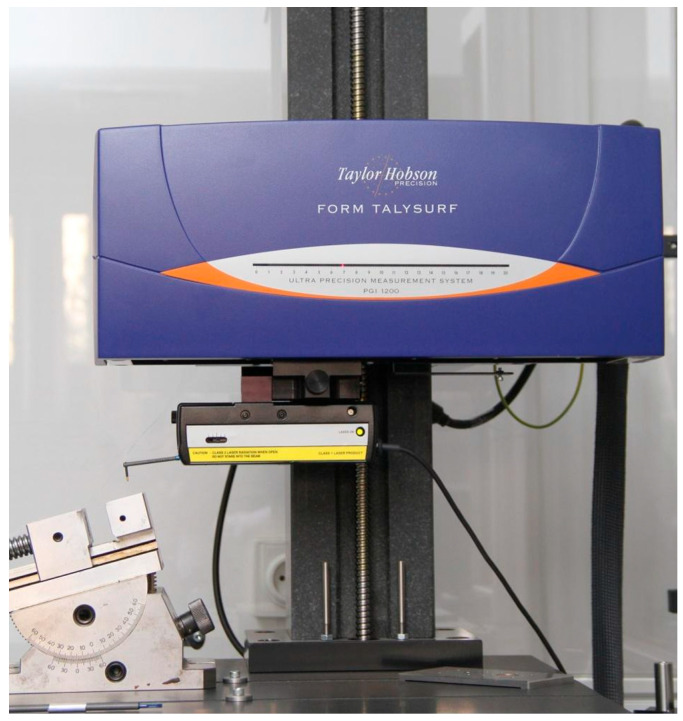
Contact profilometer used for surface measurements.

Prior to testing, the machined surface of the samples was measured using a Talysurf PGI (Figure 2) contact profilometer (Tylor Hobson, AMETEK, Inc., Berwyn, PA, USA). A measurement consisting of 200 individual measurements used to produce a 4 × 4 mm 3D isometric projection, using a Gaussian filter, was taken. Surface tests were repeated for each measurement series after finishing.

### 2.1. MEX Technology

The samples were printed on a Bambu Lab X1 Carbon printer (Bambu Lab, Shenzhen, China). The printer has a table that moves in the *Z* axis and a print head that moves in the *X* and *Y* axes. The machine’s enclosed chamber and the ability to change the temperature of the interior of the machine make it possible to print from flexible materials as well as from materials whose vapours pose a danger to human work.

### 2.2. Material

The material chosen for the tests was TPU 95A HF (Bambu Lab, China), a thermoplastic polyurethane with a Shorea hardness of 95, manufactured by Bambu Lab for high-speed printing. Selected properties of the material are shown in Table 1. The tests were carried out by the manufacturer in accordance with the applicable ISO 527 [4] and ISO 179 standards [18].

TPU 95A material has high impact strength; however, it is a material that is prone to stretching.

The properties of the material indicate that TPU 95A can also be utilised in experimental processes involving the blending of additive materials. In article [19], two ABS–TPU blends were prepared and tested to evaluate their mechanical properties. The results demonstrated that the addition of TPU leads to a reduction in tensile strength while increasing deformation and elongation. Moreover, the incorporation of TPU can alter the behaviour of ABS from brittle to ductile.

It is worth noting that the study used material adapted to high printing speeds, known as High Speed. The test samples were produced at the following speeds: top surface 200 mm/s, outer wall 150 mm/s, inner wall 250 mm/s, table temperature 35 °C, and nozzle temperature 230 °C.

### 2.3. Processing

**Figure 3:**
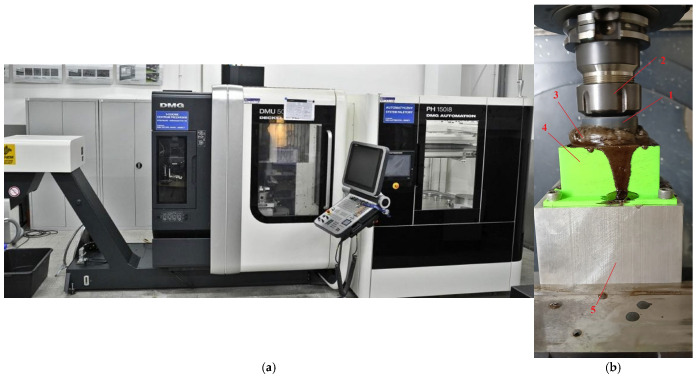
Research station: (**a**) DMG milling centre, (**b**) tool–workpiece system: 1—tool; 2—tool holder; 3—liquid; 4—workpiece; 5—workpiece holder.

**Figure 4:**
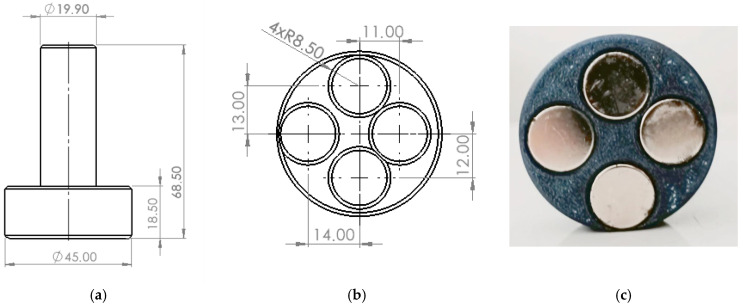
Tool design: (**a**) overall dimensions of the tool, (**b**) dimensions of the tool’s working part, (**c**) photograph of the actual tool.

The machining was carried out on a universal numerically controlled milling machine (Figure 3a) DMU 50 (DMG Mori, Tokyo, Japan). Each machining cycle lasted 3 h, with the tool rotating while remaining in a fixed position. Figure 4b presents a schematic diagram of the experimental setup: 1—tool; 2—tool holder; 3—liquid; 4—workpiece; 5—workpiece holder. In this method, the tool performs a rotational motion, while the workpiece remains stationary. No additional equipment other than that shown in the figure was employed in the study.

A ferromagnetic fluid was prepared for the test, consisting of 66.5% carbonyl iron particles, 22.5% ferromagnetic fluid EFH1, and 11% silicon carbide abrasive particles. The fluid used consisted of the following components: iron oxide (magnetite) 3–15%, oil-soluble dispersant 6–30%, and petroleum distillates (Hydrotreated Light) 55–91%. The viscosity of the ferromagnetic fluid EFH1 is 6–<12 cP.

(a) Tool

Due to the characteristics of the process, the tool shown in Figure 4 was prepared for the test. The diameter of the tool was 45 mm and completely covered the surface of the workpiece. In addition, the tool was equipped with four asymmetrically arranged sockets. Three neodymium magnets with a Br remanence induction of 1.21–1.25 T were placed in a single socket.

(b) Finishing process

**Figure 5:**
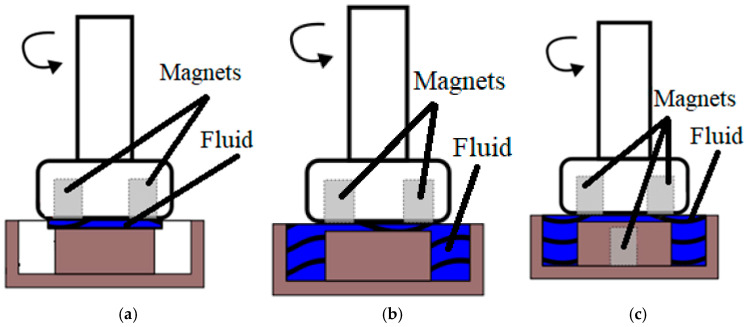
Series: (**a**) liquid applied to the surface, (**b**) full liquid, (**c**) full liquid and extra magnet.

The finishing treatment was performed in three experimental series (B, C, and D), while series A was used as the control. The corresponding process diagrams are presented in Figure 5a–c. In series B, Figure 5a, a layer of liquid was applied to the machined surface. For series C, Figure 5b, the model tub was filled with liquid above the machined surface, and in series D, Figure 5c, in addition to completely filling the machined surface, an additional magnet was fixed inside the machined surface. The tool performed 15 complete revolutions per second in a continuous 3 h cycle. There was a 1 mm gap between the tool and the workpiece surface.

## 3. Results

**Figure 6:**
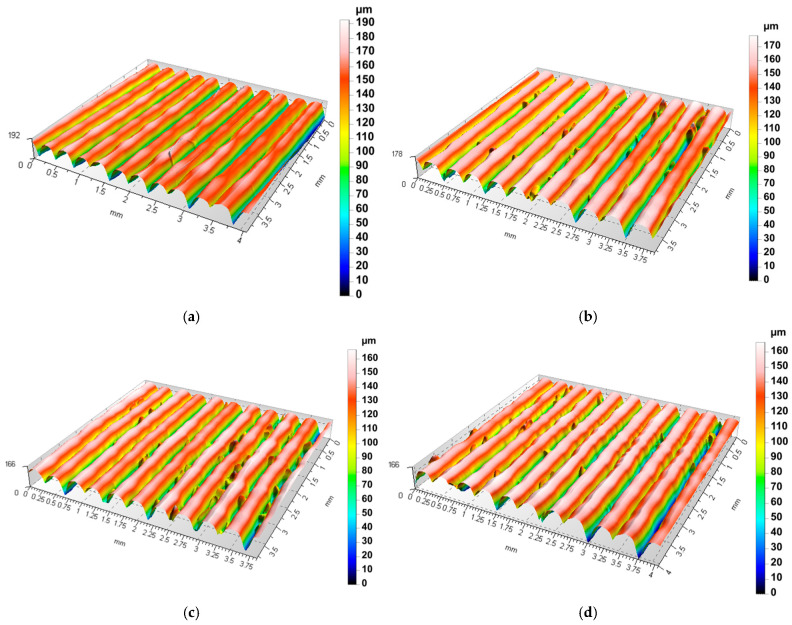
Isometric projection of selected samples: (**a**) series A, (**b**) series B, (**c**) series C, (**d**) series D.

Isometric projections of the measurement results (Figure 6a–d) are presented, showing 200 profile lines formed by connecting individual scans. Series B, C, and D were measured after machining. The control samples comprising series A were not machined and served as a reference series.

Table 2 shows the roughness results for selected samples belonging to the a–d measurement series. In the finishing process using ferromagnetic fluid, the most influential parameter is Sp (maximum peak height), whereas Sz (maximum height) represents the sum of Sp and Sv (maximum pit height). The other profile parameters indicated in the table refer to the following: Sq—root mean square height, Ssk—skewness, Sku—kurtosis, and Sa—arithmetical mean height.

The Sp parameter decreased by 31.7%, 40.5%, and 45.8% in series B, C, and D, respectively. Analysing the difference for the Sv parameter, smaller decreases can be observed than for the altitude parameter; for series B, the parameter increased by 0.2%, and for series C and D decreases of 6.7% and 5.6% were observed. Overall, decreases in the Sz parameter were observed for series B of 8.2%, series C of 15.5%, and series D of 15.8%.

**Figure 7:**
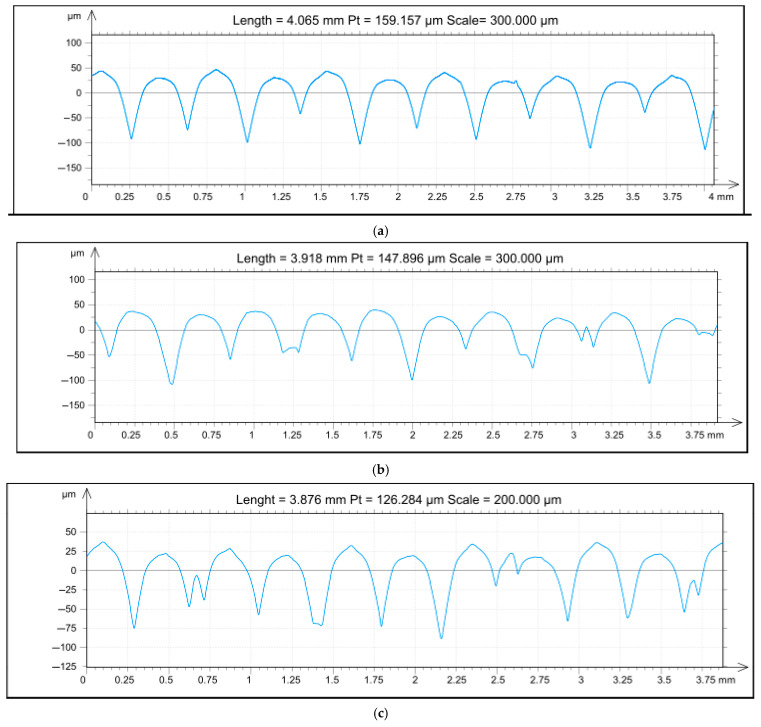

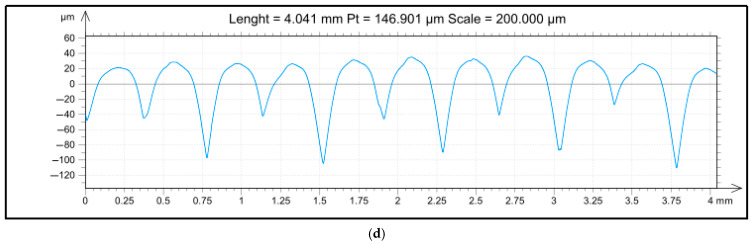
Roughness amplitudes: (**a**) series A, (**b**) series B, (**c**) series C, (**d**) series D.

Figure 7 shows selected roughness profile amplitudes for the samples (corresponding to the isometric projections of Figure 6). All profiles shown are characterised by periodicity, which could not be fully reduced by machining. The observed periodic surface roughness originates from the additive manufacturing process, reflecting the discrete elevations of the deposited plastic layers. A characteristic feature of the roughness amplitude of the TPU 95A material is the formation of visible sharp peaks at the elevations of the profile. After finishing, a smoothing of the apex surface can be observed, with this being particularly evident in the B and D series.

Table 3 shows the results of the amplitude profile roughness measurements. For series A, the parameter Pa is 27.018 μm; the highest value of the parameter was 27.089 μm for series B; and the lowest value of the parameter was 23.372 μm for series C. In the case of the parameter Pz, corresponding to the sum of the largest peak height Pp and the largest depth Pv, the smallest value was obtained for series D 125.647 μm and the largest for series B 147.896 μm.

## 4. Discussion

The solution presented is an alternative machining method using a ferromagnetic fluid with an abrasive. In the standard process, machining is carried out on machines with additional equipment in the form of an electromagnet, a ladle, and nozzles with a pump for continuous feeding of the mixture. In [11], the Ra parameter decreased from an initial value of 0.74 nm to 0.146 nm, representing a reduction of more than 80% after a treatment time of 90 min. In the case of the study, the time was extended to 3 h, the reason being the workpiece material, which is characterised by high elasticity, whereas in the article cited, silica glass, which is not a highly elastic material, was used. This suggests that in the case of materials with different hardnesses, the machining time will be different.

In the article in [17], a Ra of 81 nm was obtained; however, the sample was subjected to a manual surface lapping operation before the test was performed. Therefore, the starting surface roughness was less than the surface roughness of the samples tested in this paper.

A key aspect of liquid finishing is also the contribution of the individual components of the mixture to the total composition. In the article in [17], the research mainly focused on the respective well-balanced amount of a particular component, with the best results obtained for 25% electrolytic iron particles, 16.17% abrasives, and 58.83% water and liquid. In the case of the present study, several trials were carried out, which concluded that a percentage of 66.5% carbonyl iron particles, 22.5% ferromagnetic fluid EFH1, and 11% silicon carbide abrasive particles represented the best solution for the TPU95A material. The first sample consisted of 16.5% carbonyl iron and 25% abrasive particles, while the second consisted of 50% iron particles and 8% silicon carbide.

**Figure 8:**
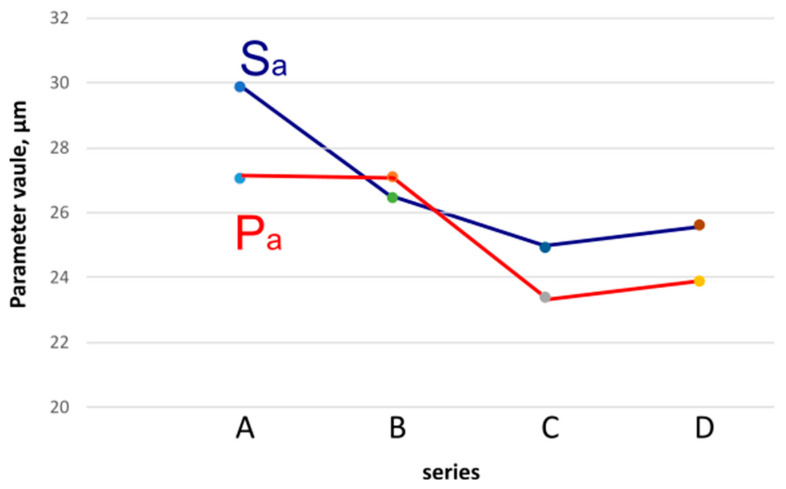
Dependence of parameters Sa and Pa.

The variation in Sa and Pa surface roughness parameters is presented in a combined graph (Figure 8).

The graph illustrates the variation in the parameters according to the indicated series. A difference between the 3D profile values can be observed, particularly for reference sample A. However, the graph indicates that the lowest values were obtained for series C.

In the cited articles, the Ra parameter decreased to values measured in the nanometre range. In the present study, using the currently employed tool and cutting process, a reduction of only 13.49% in the Pa parameter was achieved. The observed decrease in surface roughness is relatively small when compared with the reductions documented in other studies. This outcome is primarily due to the experimental design, in which the tool was assumed to perform only rotational motion. To achieve a more satisfactory effect, in subsequent trials the tool will be programmed to perform feed motions to appropriately direct the abrasive particles suspended in the fluid.

Moreover, the difference in the extent of reduction can be attributed to the differing material properties employed in the studies. In the article in [17], the machined material was PLA, which is characterised by higher hardness and lower abrasion resistance, compared with TPU 95A used in the present study. TPU 95A belongs to the class of ductile materials with low hardness, making it less susceptible to cutting-based processing. In the article in [20], the machining process of TPU 95A was also investigated. It was demonstrated that TPU 95A is highly sensitive to parameter variations and that its surface roughness cannot be predicted solely on the basis of single-factor effects. Consequently, the present study opens new possibilities for comparing the effectiveness of different processing methods.

## 5. Conclusions

In the present study, tests were carried out to reduce surface roughness, expressed by the parameters Sp, Sv, and Sz, as well as Pa and Pz. Analysis of the results led to the following conclusions:

- In the case of the Sp parameter, the largest decrease was recorded for the D series; it was about 45.8%. For this series, the smallest value of the Sz parameter was also obtained, corresponding to the sum of the largest tip height and the largest depth. This means that for series D the height of the total profile decreased.
- The C series has the smallest value of the parameter Sv, but the value of the parameter Sz is slightly higher than that of the D series. In this series, the Sa and Pa parameters also reached the lowest values.
- Analysing the amplitude of the roughness profile, series D was also characterised by the smallest value of the parameter Pz, corresponding to the sum of the largest profile height Pp and the largest profile depth Pv.
- Note that in the B series the differences between the comparison series are slightly smaller for the isometric projection parameters. For the selected 2D profile, the parameter values are larger than the control series. In subsequent tests, the solution used in the B series will no longer be considered, due to the smallest parameter difference with respect to the control samples.
- The obtained change in the parameters of the roughness profiles remained unsatisfactory compared to the BEMRF process [17]; however, the results the results provide a basis for refining the testing methodology using additional tools and for proposing adjustments to the ferromagnetic fluid composition. The analysis of the results demonstrated the potential of the developed machining method using a ferromagnetic fluid. However, the process currently requires further development. To achieve a greater reduction in surface roughness, comparable to the values reported in the studies discussed above, the following steps should be undertaken:
- Introduction of auxiliary tool motions;
- Development of an alternative tool geometry, particularly through modifications in the number of magnet slots and comparison of results with a symmetric magnet arrangement;
- Extension of the study to additional materials that are more amenable to machining, such as PLA.

The present study serves as an introduction to subsequent investigations, which aim to produce both a tool that can be implemented in a wide range of machine tools and guidelines for the processing of additively manufactured components.

## Figures and Tables

**Table 1:** Selected properties of TPU 95AHF.

Parameter	Young’s Modulus (X-Y)	Young’s Modulus (Z)	Tensile Strength (X-Y)	Tensile Strength (Z)	Elongation After Break (X-Y)	Elongation After Break (Z)	Impact (X-Y)	Impact (Z)
	9.8 ± 0.7 MPa	7.4 ± 0.6 MPa	27.3 ± 0.8 Mpa	22.3 ± 0.8 MPa	>650%	>480%	123.2 kJ/m^2^	86.3 kJ/m^2^

**Table 2:** Results of surface roughness measurements.

Parameter	Series Value A	Series Value B	Series Value C	Series Value D	Unit
Sq	36.756	32.662	30.634	32.317	μm
Ssk	−1.150	−1.174	−1.136	−1.326	
Sku	3.382	3.698	3.558	4.112	
Sp	61.291	46.541	43.637	42.047	μm
Sv	131.354	131.617	123.104	124.392	μm
Sz	192.654	178.068	166.741	166.439	μm
Sa	29.884	26.440	24.897	25.617	μm

**Table 3:** Parameters characterising the amplitudes of the roughness profile.

Parameter	Series Value A	Series Value B	Std Dev	Series Value C	Std Dev	Series Value D	Std Dev	Unit
Pp	34.136	39.692	2.706	37.452	2.796	33.292	2.268	μm
Pv	92.910	108.204	9.328	88.831	7.210	92.355	4.941	μm
Pz	127.046	147.896	10.569	126.284	7.794	125.647	5.805	μm
Pc	108.737	89.567	8.289	84.179	6.869	87.215	5.763	μm
Pt	134.294	147.896	10.569	126.284	7.794	125.647	5.805	μm
Pa	27.018	27.089	1.687	23.372	1.344	23.857	1.480	μm
Pq	32.741	33.406	1.880	28.372	1.609	29.263	1.742	μm
Psk	−1.110	−1.105	0.158	−1.024	0.087	−1.148	0.079	
Pku	3.205	3.551	0.601	3.145	0.346	3.403	0.356	

## Data Availability

The original contributions presented in this study are included in the article. Further inquiries can be directed to the corresponding author.
